# The Mediator CSR Plays the Effective Leadership Belief Role for Resource Dilemma Handling Leadership in Organizational Commitment During Sustainability Development

**DOI:** 10.3389/fpsyg.2022.874646

**Published:** 2022-06-13

**Authors:** Kuo-Hua Chan, Shang-Ping Lin, I-Tung Shih

**Affiliations:** ^1^Department of Business Administration, National Yunlin University of Science and Technology, Yunlin, Taiwan; ^2^Department of Business Administration, Chaoyang University of Technology, Taichung, Taiwan

**Keywords:** Resource-Dilemma-Handling Leadership, sustainability, transformational leadership, corporate social responsibility, organizational commitment, Effective Leadership Belief, mediator

## Abstract

The authors aim to explore a better fitting leadership style that is designed for the sustainable era in believing and committing to work for cherishing resources and developing the organization toward a new sustainable direction. This study developed the questionnaire items of the Resource-Dilemma-Handling-Leadership (RDHL) scale, representing a new sustainable era's new leadership style, and then to compare it with the transformational leadership style in order to highlight the importance of RHDL for sustainable development. This study took companies, which have more than 100 employees in Taiwan as research samples. Those companies were selected because they were socially tagged as being operating continuously for more than 20 years and identifying themselves with the operational orientation of social responsibility as their business philosophy and core values for management. A total of 532 valid questionnaires were collected, with a 90.6% valid return rate, and tested with the SEM method. Consistent with the authors' inferences from the literature, the test results suggest that CSR plays the role of full mediator between RDHL and OC. CSR itself is like a sense of responsibility, giving employees a sense of mission, to complete meaningful sustainability-relevant tasks in the organization. RDHL, compared with TL, has a better prediction power on CSR and OC. Theoretically, this study implies that the impact of leadership of the organization on OC in the aspects of sustainable development should be going through the influence of the ELB system in the form of CSR to promote the organization's internal and external organizational CSR performance, with added internal strengthening power from OC. Practically, the new RDHL concepts brought up by this study include the training and enhancement of leadership skills based on the content of the scale items being explored. The new RDHL scale contains a comprehensive description of the spirit of the new sustainable era's leadership style. Also, the future applications of RDHL ideas in the form of human resource development should help the realization of the ideally sustainability behavioral patterns of leaders and employees in the organizations.

## Introduction

The key to achieving organizational sustainability is CSR practice in the organization (Setthasakko, 2007). Sustainable development does not simply mean that something can be sustained, which involves developing innovative measures that maintain environmental balance and harmony in a way that does not harm the development of others in the present and future environment (Hargreaves and Goodson, 2004). According to Brundtland Commission, the definition of sustainable development is “development which meets the needs of current generations without compromising the ability of future generations to meet their own needs” (Kono, 2014). This definition covers both systems within and out, including institutions, organizations, government, etc.

In a rapidly changing and challenging global marketplace, leaders should pay attention to social and ecological issues (McCann and Sweet, 2014). The Global Reporting Initiative's (GRI) Global Sustainability Report includes three main dimensions: environmental, economic and social. By promoting CSR, companies can clearly understand how to achieve sustainability (Global Reporting Initiative, 2006) and appropriately integrate sustainable development into their operations due to the involvement and commitment of external stakeholders outside the organizations. That is because the stakeholders do not only focus on the profitability or benefits of companies. They also pay attention to relevant issues of sustainable development from the external perspective: the real practice of CSR through the pulling power sourcing from the external stakeholders. Therefore, it is appropriate that this study used CSR as a variable to examine the impact of leadership on sustainability performance outside the organization. Therefore, this article extensively contributes to explaining the Effective Leadership Belief (ELB) (Rus et al., 2010) concept with the variable of CSR. If CSR is viewed as an ELB, employees' recognition of CSR beliefs will become an important mediator for promoting organizational commitment (OC). This may have indirectly proved that employees are willing to invest in OC because they identify with the leader's effective beliefs.

In short, leaders have played a critical role in developing sustainability. However, insisting on the sustainable development of organizations is not an easy mission, especially when profitability is the general beneficial goal of the stakeholders of the organization. This study aims to identify a new leadership style that cares about the new generation's sustainable development and the profitable goals set up by the stakeholders in such a resource-lacking era. That is to say: leaders should deal with the needs of sustainability of the current generation and still have the ability to handle the needs of the next generation.

In the process of sustainable leadership, the role of the leader is to: maintain co-development with others (Hargreaves and Goodson, 2004) and to make employees feel more valued for their presence and therefore enjoy playing an important role in the development of the organization (Sharma et al., 2019). Leaders and their partners and associates construct a network based on shared values, affection, trust, commitment and energy, which shares the same values as the function of the team's actions. This is conducive to and makes a critical impact on the sustainability of the organization (Horlings and Padt, 2013; Kurucz et al., 2017) through the distribution of cognitive principles, ideas, and beliefs to employees, co-workers, partners, and the like (PSI model), and the like (Fan et al., 2021). Employees' perceptions of organizational sustainability are influenced by their recognition of and participation in the organization's cognitive principles, ideas, and beliefs disseminated by leaders (Tilleman, 2012).

Among all those organizations, sustainable systems, norms, cultures, etc., are influenced by leaders' management. More than four possible leadership styles have been categorized in the past: authoritarian, participative, delegative, transactional, and transformational. Among these leadership styles, the transformational leadership style has been frequently indicated as the possible candidate for the leading sustainable development of organizations. However, in a time of rapid change, the leader and his beliefs become important. The leaders of the new generation need not only have competition, skills, and well perception of cherishing resources and efficient resource utilization. This kind of recognition is very special because human beings need beliefs and a sense of responsibility beliefs in cherishing resources. Therefore, the authors of this article, according to the literature review and interviews with the 5 seniors who have worked in the companies that won the sustainable management award in Taiwan for more than 25 years, developed the items of the Resource-Dilemma-Handling-Leadership (RDHL) scale, which is better suitable for the new century to highlight the importance of leadership for sustainable development. RDHL leadership focuses on leaders' competencies in creating organizational sustainability through integration or reform in the face of rapid changes and unpredictable challenges in the future, and also on personalized management of employees and maintaining the collectivized and sustainable development of the whole organization to address the dilemma between individuals and the collectives. The most important significance and capability of leadership is the ability to create a sustainable future for the organization through leadership integration or reform in the face of rapid changes and unpredictable challenges in the future, as principles suggested by Efthimiou (2017).

In terms of the impact of leadership and organizational sustainability, transformational leadership (TL) is the most frequently applied theoretical framework in past studies (Choi, 2016; Tabassi et al., 2016; Jiang et al., 2017). Also, scholars had used the SEM model to study TL's direct and indirect significant effects on organizational performance and found that leadership combined with CSR will lead to high performance (Khan et al., 2018). But, there were also some research indicated the new generation of leaders needs to have competition, skills, and well perception of cherishing resources being utilized in sustainable development for the new century (McCann and Sweet, 2014; Suriyankietkaew and Avery, 2016; Lukoschek et al., 2018). Leaders' practice of CSR provides a basis for organizational sustainability (Allen et al., 2017). Leaders' practice of CSR will form the core of sustainable leadership management because leadership positively impacts organizational commitment (McMurray et al., 2010). CSR brings a sense of mission to employees feel more meaningful and fulfilled the tasks assigned in the organization, so that OC is generated (Wang et al., 2019). Based on prior research background, the main purpose of this study is to examine both TL & RDHL on CSR, and the mediation effect on OC, in such a requesting-sustainable-resources era, and further explore the impact of should be adopted through the influence of leadership on the external organizational performance of CSR to promote the internal strengthening of OC, The training and enhancement of leadership skills can be improved based on the impact of RDHL in the future application of human resource management strategies and the training and succession of leaders, in order to create and achieve the goal of organizational sustainability.

In short, the authors aim to explore a better fitting leadership style that is designed for the sustainable era in believing and committing to work for cherishing resources and developing the organization toward a new sustainable direction. Theoretically, this study uses the theories of OC in the aspects of sustainable development, ELB in the form of CSR, to generate a new sustainable leadership style, RDHL, and tests RDHL's effectiveness with TL in order to offer an instrument to organizations in promoting organizational sustainability. The practical aspects derived from the new RDHL scale items' concepts have integrated the ideas of sustainable leadership skills and style, which helps the realization of the ideally sustainability behavioral patterns of leaders and employees in an organization. And finally, the RDHL scale was presented to be well developed with qualified psychometrics evidence.

## Literature Review

### Resource-Dilemma-Handling-Leadership

#### Attributions of Resource-Dilemma-Handling-Leadership

Achieving organizational sustainability requires an overview of the past, present, and future, so to overcome the dilemmas or difficulties faced, leadership needs to consider the organization's long-term sustainability and short-term survival needs. According to the literature review, this study summarized the following attributions, traits and concerns of RDHL.

1. It is often difficult for an organization to achieve efficiency and spur innovation concurrently. Under leadership behaviors, an organization encourages innovation while ensuring its efficiency to achieve sustainable performance (Lukoschek et al., 2018). The above research construct was included as an item of this study: “Executives make long-term and substantial investments to support employees' innovative behaviors while boosting short-term operational efficiency of the company.”
2. An organization consists of many members, and their time diversity and perspectives vary. When the time diversity of the members is low, leaders can easily manage conflicts of time perspectives, but as the time diversity increases, the differences in time perspectives may drive up conflicts. If managed well, conflict resolution can facilitate knowledge sharing for sustainability among members (Najam et al., 2018). The above research construct was included as an item of this study: “Executives value their own time and cost-effectiveness while devoting themselves to internal communication and the sharing of information, despite the high time cost.”
3. Generally speaking, adopting diversity and multiculturalism in organizations may reduce efficiency and increase management costs, thereby affecting performance. It has been shown that diversity management and multiculturalism can reduce differences in an organization and improve its sustainability performance (Dreachslin et al., 2017). The above research construct was included in this study: “Executives attach great importance to cost-efficient management of personnel while paying heavy personnel costs on diverse employment and management, such as employees' gender, age, race, nationality, and religion.”
4. There are often conflicts and contradictions between business/public interests and values. Studies have suggested that organizational sustainability can create long-term well-being and value for all stakeholders through leadership or management processes, thus, fueling the sustainable growth of corporate profits (Suriyankietkaew and Avery, 2016). The above research construct was included as an item of this study: “Executives value the interests and well-being of the public without compromising the interests of the company”.
5. There is often a gap between social values and corporate performance. Some studies have indicated that organizational sustainability must be created by strategies for leadership integration to balance the support from social, physical, ethical, and business practices, and also by incorporating emphasis over and attention to environment and society as well as securing innovations in technical or business process to ensure optimal organizational performance (McCann and Sweet, 2014). The above research construct was included as an item of this study: “Executives value the company's profitability while spending on environmental protection, despite the heavy cost of capital.”

To ensure the information mentioned above for RDHL is correct (validity concerns), the authors interviewed five senior executives with more than 20 years of experience in companies established for more than 25 years and ever received the Taiwan Corporate Sustainability Awards. Those top management team members had offered valued opinions about how their leaders had “properly managed” and “balanced” their business management and decision-making as an RDHL (Please see summary below). (Note: interview data was available upon request). Based on the literature and interview results, this study developed the RDHL scale (Appendix A). Moreover, the authors use RDHL as one of the variables for quantitative validation and testing in this study.

1. Executives place a high value on the financial performance of the company's operations while helping employees develop their careers.
2. Executives attach great importance to creating the company's interests while sharing the business results with employees.
3. Executives are “severe with themselves” and “lenient with others.”
4. Executives value the company's identity and niche while respecting and adopting employees' viewpoints.
5. Executives emphasize building and preserving the company's traditional culture while leading the company to pursue constant breakthroughs and innovations.
6. Executives invest heavily in enhancing the company's core business strengths while spending heavily on the employees' physical, mental and spiritual growth and care.
7. Executives attach great importance to improving the company's performance while providing consumers with more open and transparent information on product trading.
8. Executives closely work with the government to limit statutes and promote policies while creating the company's operating income.

### Organizational Commitment

In realizing the sustainable development of the organization, when faced with the dilemma or difficulty of the external performance of the organization, the leader's performance is more likely to obtain external attention and recognition. Therefore, the impact of CSR embedded in leadership will be important in achieving the goal of creating and achieving organizational sustainability.

RDHL basically affects three dimensions of OC, namely, Affective Commitment, Continuance Commitment, and Normative Commitment (Meyer and Allen, 1997). OC is affected by employees' perceptions of the organization's environmental sustainability (Tilleman, 2012), and leadership affects the OC of employees' internal energy of the organization, thus, attaining the goal of creating organizational sustainability. In the process of achieving organizational sustainability, leaders, when faced with dilemmas or difficulties external performance of the organization, can increase the impact of employees. OC in order to fulfill the goal of creating and achieving organizational sustainability, whether inspiring employees with values and concepts or taking concrete implementation plans and measures.

Previous studies have proved the correlation between employees' perceptions of CSR and employees' OC behaviors (Paruzel et al., 2021). As corporate sustainability entails the involvement and commitment of external stakeholders, companies can get a clear perspective of achieving sustainability by promoting CSR (Global Reporting Initiative, 2006). Therefore, the promotion of CSR requires the recognition of interactions with consumers, from the side of employees (Glavas, 2016; Jones et al., 2017a), external parties, and even stakeholders at the micro-level, which can also be extended to cover the recognition of community development, social issues, and overall economic issues at the macro level.

In achieving organizational sustainability, when faced with dilemmas or difficulties external performance of the organization, leaders are more likely to gain external attention and recognition for their performance in implementation. As a result, the impact of CSR will be enhanced mainly by taking implementation plans and measures made by RDHL to fulfill the goal of creating and achieving organizational sustainability.

### CSR as the Effective Leadership Beliefs for RDHL to Promote Sustainable Development

Employee perceptions of a leader's beliefs are related to ratings of the target leader's performance (Weber et al., 2018). Leader categorization theory suggests that leaders have behavioral schemas that pertain directly to the leader role and that these schemas represent a foundation for the generation of behaviors (Leader categorization theory; Meindl and Ehrlich, 1987; Lord and Maher, 1993; Rus et al., 2010; van Gils et al., 2010).

Not all leaders will strongly care about their effective leadership beliefs (ELBs). Previous studies have identified about certain effective leadership beliefs; effective leadership beliefs are suggested to be related to role-related schemas, which are like behavioral guides to the leaders (internal perspective) and their employees (external perspective) (Rus et al., 2010).

Therefore, it is important to identify an effective leadership schema and its associated effective leadership beliefs. In this case, the effective leadership beliefs that the authors identify are CSR. The role of CSR has influenced the subordinates' observation and perception of their leaders' behaviors, internal beliefs, and relevant performance.

Rus et al. (2010) ever explored the content of effective leadership beliefs (self- vs. group serving) and the way of leader resource allocations. However, the associative mechanism between Effective Leader Beliefs and Schema is not detailed described in this 2010 paper till 2021, in which Fan et al. presented the PSI model to describe the relationship between principles, self-efficacy, and insisting on mental toughness *via* Schema theory. The PSI model Fan et al. (2021) indicated that people has used schema to appraisal events confronted and schema is the existing, beliefs, assumptions and unspoken assumptions that people infer meanings in a concrete manner (Fan et al., 2021). That means employees will observe leadership's opening behaviors and speeches implicitly indicate the content of their emphasized values and beliefs (it is CSR in this case) embedded in their schema categories.

The authors of this study suggested that CSR itself is like a sense of responsibility, which brings a sense of mission to employees. Because of the importance attached to the sense of mission, employees feel more meaningful and fulfilled the tasks assigned in the organization, so that OC is generated. This is like a hatchery of employees in learning meanings of mission and a sense of achievement when completing meaningful tasks in the organization. For this study, through the CSR's hatchery in multiple emotional senses, employees are regarding CSR as ELB during the process of sustainable development. Nevertheless, this could be more prominent in NPO organizations. Please see illustration of the concept of hatchery in **Figure 3**.

For example, Ferrari (2004) completed measures on community self-efficacy, sense of community, and caregiver satisfaction and stress in Australia and found respondents experienced a relatively strong sense of common mission and a stronger sense of reciprocal responsibility to help their peers. Moreover, Schnaider et al. (2009) interviewed 21 family care providers to identify and learn the meaning of being a family care provider, and the following representations were identified about the event of being a care provider: “to provide care,” “affection and responsibility,” “act of love,” “mission and vocation,” “obligation” and “difficulty.”

The key to achieving organizational sustainability is incorporating CSR into the organization's strategy and practice (Setthasakko, 2007). As stated in the Global Reporting Initiative (GRI), the report on global sustainability covers three dimensions: environment, economy, and society. Since a company's sustainability is related to the involvement and commitment of external stakeholders, a company can gain a clear idea of how to achieve sustainability by promoting corporate social responsibility (CSR) (Global Reporting Initiative, 2006). Since there are economic, social and environmental dimensions in sustainable development, an organization must consider the sustainability impact of its CSR on the stakeholders (Çalişkan, 2015). Leadership plays a vital role in the corporate responsibility of the organization (Mazutis and Zintel, 2015).

Organizational commitment (OC) refers to the recognition and involvement of employees within an organization, and it is affected by their perception of the organization's environmental sustainability (Tilleman, 2012). Leadership is the prerequisite for employees' OC (Haque et al., 2021). Leadership can encourage the collaboration between members of the organization and between different departments and promote the organization's sustainability (Avissar et al., 2018). When a leader helps employees realize a higher learning culture and achieve higher work complexity, employees will demonstrate higher OC (Joo and Lim, 2009). When the leadership can express visions, improve team goals and stimulate intelligence, employees also demonstrate higher OC (Joo et al., 2012). When the leadership is developing a good strategy for sustainable development, a consistent commitment to sustainability within the organization is required (Lee and Schaltegger, 2014). Leadership plays an important role in promoting CSR (Fenwick, 2007; Mayo et al., 2016; Mallén Broch et al., 2020; Nguyen et al., 2021).

**Figure 1:**
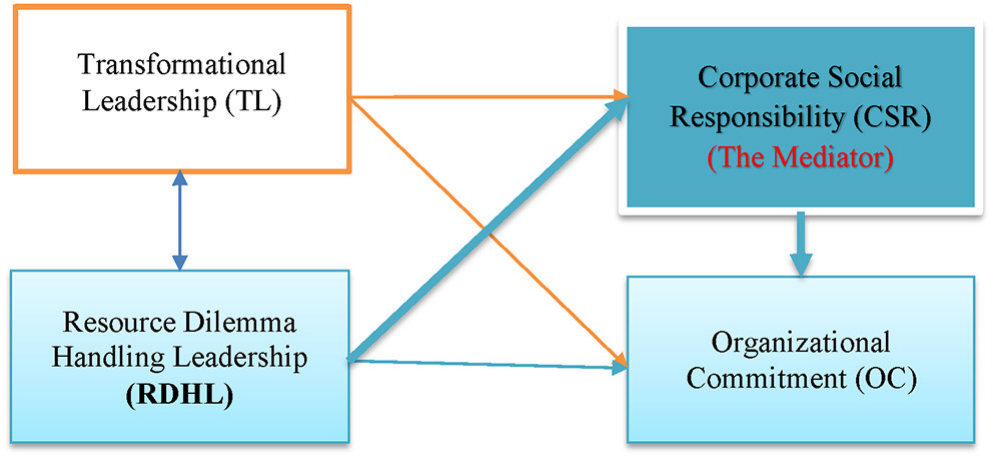
Research framework of this study (The key mediating role of CSR in helping RDHL to promote the level of organizational sustainability).

That is to say, to help find ELB such as CSR in this study in enhancing organizational citizens' organizational commitment to promoting sustainable development of the global village becomes one significant task of the scholars. Therefore, this study adopts CSR as a variable to explore the impact of leadership on the external sustainability performance of the organization. In achieving organizational sustainability, we assume RDHL is positively and significantly associated with CSR, which should play the key role of mediator in promoting leaders' (RDHL) leading and committing to the organization (OC: organizational commitment). (Please see Figure 1, the conceptual framework of this study).

### Organizational Sustainability and Sustainable Leadership

The term sustainable development was originally used to explain theories that concerned economic development, ecological environment and social equity (Harris, 2003). The importance of leadership to organizational sustainability is foreseen. Nevertheless, the perspective of sustainable development does not simply mean that something is sustainable; it also involves developing innovative measures without compromising the development of others in the present and future environment (Hargreaves and Fink, 2000). Bendell et al. (2017) used the term sustainability as an abbreviation for sustainable development, arguing that sustainable leadership begins with sustainability and emphasizing that achieving sustainable development is a critical decision that sustainable leadership makes when confronted with organizational dilemmas.

Organizational sustainability could result from the influence of leadership, which creates long-term well-being and sustainable value for all stakeholders. The leadership focuses not only on social and environmental responsibilities but also on spurring profitable growth and achieving a company's sustainability (Suriyankietkaew and Avery, 2016). One of the key forces influencing organizational change or sustainable development, in the long run, is leadership (Hargreaves and Goodson, 2004). Leadership's greatest value stimulates organizational sustainability and better performance (Shriberg and MacDonald, 2013).

For this reason, to properly face and handle organizational dilemmas, leaders should be able to strike a balance between the current continuous survival needs of the organization and its sustainable development. Based on the above literature concerning the influence of leadership on organizational sustainability, it was found that organizations are often confronted with the dilemmas of conflicts and contradictions throughout their present and sustainable development (e.g., McCann and Sweet, 2014; Suriyankietkaew and Avery, 2016; Dreachslin et al., 2017; Lukoschek et al., 2018; Najam et al., 2018).

Organizational sustainability must be created by strategies for leadership integration to balance the support from social, physical, ethical, and business practices, and by incorporating emphasis over and attention to the environment and society and securing innovations in technical or business processes to ensure optimal organizational performance (McCann and Sweet, 2014). Furthermore, the dilemmas faced by leadership are sometimes chronological or sometimes, perhaps concurrent. The most important significance and capability of leadership is the ability to create a sustainable future for the organization through leadership integration or reform in the face of rapid changes and unpredictable challenges in the future (Efthimiou, 2017). As leadership is one of the key forces for long-term organizational change or sustainability (Hargreaves and Fink, 2004), its greatest value is facilitating and maximizing the sustainability of organizations (Shriberg and MacDonald, 2013). In the development of organizations, leaders often face sequent or co-existing conflicts and contradictions. The most important meaning and leadership can integrate or change leadership to create sustainable outcomes and future sustainability in the face of rapid change and unpredictable challenges (Efthimiou, 2017).

Organizational sustainability results from leadership, which creates long-term well-being and sustainability value for all stakeholders and is concerned with social and environmental responsibility and creating profitable growth and achieving sustainability (Suriyankietkaew and Avery, 2016). When an organization attaches importance to employees' freedom, trust and autonomy, the leadership should focus on personalized management of employees and maintaining the collectivized and sustainable development of the whole organization to address the dilemma between individuals and the collective (Taskin and Devos, 2005). To achieve and create organizational sustainability, the concept of “Resource-Dilemma-Handling Leadership of Sustainability” has been proposed in previous studies, but concrete measurement tools for empirical research are still lacking. In previous research, though many types of research have focused on the importance of RHDL for organizational sustainability (McCann and Sweet, 2014; Suriyankietkaew and Avery, 2016; Dreachslin et al., 2017; Najam et al., 2018), those works are mostly case or qualitative studies and lack relevant empirical validation due to the lack of appropriate measurement tools. Therefore, this study constructed an RHDL measurement tool to further compare and contrast with the most frequently used transformational leadership theory in past research, which means testing RDHL & TL together in an association model for finding significant indicators of sustainable-type of leadership for organizations.

### The Difference Between TL and RDHL and Sustainability (The Need for Building up RDHL)

In terms of the impact of leadership and organizational sustainability, transformational leadership (TL) is the most frequently applied theoretical framework in past studies. TL has positively influenced employee sustainability performance (Jiang et al., 2017). TL also has found a direct impact on the achievement of OC (Tabassi et al., 2016; Palalic and Ait Sidi Mhamed, 2020). Through OC, leadership style directly or indirectly affects social and environmental performance (Patiar and Wang, 2016) and can be used to respond to future challenges and promote the sustainable development of organizations (Jones et al., 2017b). Some scholars have used the SEM model to study TL's direct and indirect significant effects on organizational performance and found that leadership combined with CSR will lead to high performance (Khan et al., 2018). Therefore, leaders' practice of CSR provides a basis for organizational sustainability (Allen et al., 2017). Leaders' practice of CSR will form the core of sustainable leadership management because leadership positively impacts employee commitment and organizational evaluation (McMurray et al., 2010).

It is often difficult for organizations to promote efficiency and innovation together. Thus, leadership encourages innovation while ensuring organizational efficiency is necessary to achieve sustainable performance (Lukoschek et al., 2018). As organizations consist of many members who have diversified schedules and senses of time, well leadership and conflict resolution can facilitate sustainable knowledge sharing (Najam et al., 2018). In general, applying diversity and multiculturalism in an organization's management may reduce efficiency and increase management costs, affecting performance. Where there is often a conflict between business interests and the public good, leadership can create long-term well-being and values for all stakeholders, thereby contributing to the sustainable growth of corporate profits (Suriyankietkaew and Avery, 2016). There is often a real gap between social values and corporate performance, which can be integrated through leadership management that combines social, physical, ethical and business practices with environmental and social concerns and technological or business process innovations to ensure optimal organizational performance for sustainable growth (McCann and Sweet, 2014).

Strategic sustainability requires collaboration between leaders, employees, and the top management team's organizational commitment, with the glue of culture, values, and ethics (Landrum and Edwards, 2012), which are extended from CSR beliefs to achieve the final goal of corporate performance and increase of competitiveness. This study is embedded in an investigation of the individual level's organizational commitment toward CSR and sustainability behavior; thus, organizational commitment is assigned as a variable in investigating organizational individuals' commitment levels.

In previous studies, it has been found that a mediating relationship between CSR and OC and that employees' perceptions of CSR are relevant and influential in terms of employee trust, organizational identity and OC (Farooq et al., 2014). Employees' recognition of CSR and organizational trust also has a significant indirect effect on OC (George et al., 2020). Therefore, there is a mediating role between CSR and OC, and leaders should consider CSR as an investment rather than a cost in creating and achieving organizational sustainability (Gupta, 2017). Thus, this study used CSR as a mediator between TL, RHDL and OC.

## Research Methods

### Research Subject

#### Sample Data Analysis

This study took companies with 100 or more employees in Taiwan as samples. A total of 587 questionnaires were collected, of which 532 were valid. A valid return rate of 90.6%. To balance the differences in leadership perceptions between senior executives and junior employees, top management teams and employees accounted for 50% of the target population for the questionnaire. These sample companies have been operating continuously for more than 20 years and contain the core value of social responsibility in their business philosophy for management. Among them, 10 (37.0%) are manufacturing companies in Taiwan, such as semiconductors, electronic technology and plasticization, construction and food processing. Among the leading enterprises, 17 (63.0) % of the service industry are leading enterprises Taiwan service industry, such as shipping, catering, finance, information services and intermediary services, with more than 100 to < 200 employees 5 (18.5%), 9 (33.3%) with more than 200 to < 1,000 employees, and 13 (48.2%) with more than 1,000 employees. Questionnaire sample data, 271 males (50.9%), 261 females (49.1%); 252 (47.4%) aged 20–39 years old, 280 (52.6%) aged above 40–65 years old, education level 99 people (18.6%) from high school to junior college, 295 people (55.5%) from universities, 138 people (25.9%) from research institutes (inclusive); 263 people (49.4%) are currently working as senior executives of the company, 269 people are grassroots cadres and employees (50.6%).

### Research Tools (Scales)

The questionnaire was developed with five parts that included (1) basic personal information (gender, age, education, current job title, industry type, company employees, and whether they hold executive positions), (2) a TL scale, (3) a RDHL scale, (4) an OC scale, and (5) a CSR scale, all of which were developed in accordance with a five-point Likert Scale (from 1=strongly disagree to 5=strongly agree). Apart from the basic information, the other scales were designed as follows.

#### Resource-Dilemma-Handling-Leadership Scale

According to a literature review (McCann and Sweet, 2014; Dreachslin et al., 2017; Lukoschek et al., 2018; Najam et al., 2018) and interviews of five senior executives with more than 20-year working experience in companies who have received an over-25-year awards for sustainable business in Taiwan, this study summarized and constructed a comprehensive new RDHL consisting of 13 items (Appendix A).

After the RDHL scale was administered, exploratory factor analysis was conducted to extract factors by the principal component analysis and common factors by varimax. The analysis resulted in a significant level of Bartlett's spherical test (*p* < 0.05), and a KMO value of 0.94 (>0.50) was found upon examination, indicating that the study data were meritorious for factor analysis (Kaiser, 1974). The items were then deleted according to the construct validity of the deletion criterion, such as those with factor loadings <0.5 or those with unclear factor affiliation. Two constructs were extracted: four questions on “Resolving dilemmas based on values and leadership strategies” and six questions on “Resolving dilemmas based on execution and management operations”. The cumulative explanatory variance was 72.76%.

#### Transformational Leadership Scale

The TL scale used in this study was selected from the MLQ-5X scale developed by Bass and Avolio (2000), consisting of 20 questions. The constructs and questions included: 8 questions on idealized influence, 4 questions on inspirational motivation, 4 questions on intellectual stimulation, and 4 questions on individualized consideration.

#### Corporate Social Responsibility Scale

In this study, the CSR scale was based on Hwang and Chi (2005) recommendations on CSR measurement. As corporate sustainability involves external stakeholders' participation and commitment, companies can clearly understand how to achieve corporate sustainability by promoting CSR (Global Reporting Initiative, 2006). However, CSR strategy involves micro-level interactions with consumers, facilities, employees and external groups, as well as shareholders and creditors, and can be extended to macro-level issues such as social issues, community development, and even national, social and economic aspects. Therefore, according to the results of the exploratory factor analysis, the scale was divided into two constructs: 7 questions on micro-level CSR and 6 questions on macro-level CSR. The cumulative explanatory variance was 68.23%.

#### Organizational Commitment Scale

In this study, OC scale was used. After exploratory factor analysis, 3 questions on affective commitment, 3 questions on continuance commitment and 4 questions on normative commitment were extracted. That were consistent with the three constructs of scale (Jaros, 2007). The cumulative explanatory variance was 72.90%. In addition to applying the TL scale developed by Bass and Avolio (2000) the summary tables of factor and reliability analyses for the other three scales are shown in Table 1.

**Table 1:** Summary table of factor and reliability analyses of the scales.

	**Constructs**	**Cronbach's α**	**Overall reliability factor**	**Cumulative explanatory variance**
RDHL scale	Resolving dilemmas based on values and leadership strategies	0.869	0.939	72.76%
	Resolving dilemmas based on execution and management operations	0.934		
CSR scale	Micro-level CSR	0.925	0.944	68.23%
	Macro-level CSR	0.896		
OC scale	Affective commitment	0.840	0.877	72.90%
	Continuance commitment	0.793		
	Normative commitment	0.820		

## Analysis

### Demographic Statistics

The target population of this study was Taiwan's joint-stock companies with 100 or more employees, of which 10 (37.0%) were in the manufacturing industry, 17 (63.0%) in the service industry, 5 (18.5%) with more than 100 to < 200 employees, 9 (33.3%) with more than 200 to < 1,000 employees, and 13 (48.2%) with more than 1,000 employees. According to the data of the participants, 271 (50.9%) were male, and 261 (49.1%) were female; 252 (47.4%) were aged 20 to below 39, 280 (52.6%) were aged above 40 to below 65, 99 (18.6%) were educated from high school to college, 295 (55.5%) were university students, and 138 (25.9%) were graduate students or above. 25.9%; 263 (49.4%) were senior executives, and 269 (50.6%) were junior executives and employees.

According to West et al. (1995), the normality test mainly examines the distribution of variables. The value of each variable must be < 2 according to its standard. Otherwise, the variable is considered as an extremely skewed distribution, and the kurtosis must not be > 7; otherwise, it is not by the normality assumption. The analysis showed that the kurtosis ranged from −0.682 to 1.516, so the measured values did not violate the assumption.

The statistical analyses of the variables (Table 2) indicated that the population mean of TL is 3.96 and that employees have a high level of agreement to the values and beliefs mentioned by their supervisor (top leader) that he/she considers most important. The highest level of leadership perceived by TL is idealized influence (M = 4.01, SD = 0.59), followed by inspirational motivation (M = 3.95, SD = 0.72), intelligence (M = 3.95, SD=0.72), intellectual motivation (M = 3.92, SD = 0.70), and personalized care (M = 3.91, SD = 0.76) at the last.

**Table 2:** Summary table of descriptive analyses of each question and construct.

**Scale**	**Construct**	**M**	**SD**	**M**	**SD**
TL scale	Idealized influence	4.01	0.59	3.96	061
	Inspirational motivation	3.95	0.72		
	Intellectual stimulation	3.92	0.70		
	Individualized consideration	3.91	0.76		
RDHL scale	Resolving dilemmas based on values and leadership strategies	3.61	0.75	3.69	0.68
	Resolving dilemmas based on execution and management operations	3.74	0.69		
CSR scale	Micro-level CSR	3.96	0.73	3.90	0.61
	Macro-level CSR	3.83	0.79		
OC scale	Affective commitment	3.94	0.72	3.71	0.63
	Continuance commitment	3.58	0.74		
	Normative commitment	3.64	0.68		

The population mean of RDHL is 3.69, showing that employees have a moderate to high level of agreement that their supervisors (top leaders) will be highly responsive to governmental restrictions and policies, while at the same time creating operational benefits and performance for the company. The highest level of leadership perceived by RDHL is Resolving dilemmas based on execution and management operations (M = 3.74, SD = 0.69), followed by Resolving dilemmas based on values and leadership (M = 3.61, SD = 0.75).

The population mean of CSR is 3.90, indicating that employees believe that their company's responsibility to shareholders is to pursue sustainability and maximize corporate value, with micro-level CSR (M = 3.96, SD = 0.61) scoring the highest, followed by macro-level CSR (M = 3.83, SD = 0.70). The population mean score of OC is 3.71, indicating that employees felt happy to be part of the company and had a medium to high level of agreement with the organizational commitment of the company they were working for, with affective commitment (M = 3.94, SD = 0.72) having the highest level of agreement, followed by normative commitment (M = 3.64, SD = 0.68), and continuance commitment (M = 3.58, SD = 0.74).

### Goodness of Fit Analysis

This study used the AMOS 26 statistical software to conduct a SEM pattern analysis to verify the fit of TL, RDHL with CSR and OC. The test was conducted with descriptive statistics to examine the data collected for normality test and analysis, and fit indicators of the structural equation model were used to test the leadership model and explore the causal relationships between potential variables and the hypothetical fit of the model. Finally, structural relationships were investigated based on the measurement of model path coefficients.

In this study, the structural equation modeling fit, referring to Hair et al. (2009), was assessed by three indicators, namely absolute fit index (RMR <0.05, GFI >0.90, RMSEA <0.08), relative fit index (CFI >0.90, NFI >0.90) and parsimonious normed fit index (PNFI >0.50, χ2/df < 5, CN > 200). The three measures were used to assess the overall goodness-of-fit, which evaluate whether the theory can account for the actual observations.

For the assumptions of structural equation modeling, the offending estimates were used as the basis for the model fit test. The variables should be first examined to meet four requirements; then the overall model can be discussed and analyzed (Hair et al., 2009).

The analysis showed that all the variables in this model were positive, with standard errors (SE) ranging from 0.007 to 0.035 and standardized coefficients of estimation >0.95, all of which were significant at >0.001, thus meeting the four requirements suggested by Hair et al. (2009). This means that the model has no violation of the estimation and a final discussion and analysis can be implemented. The results in Table 3 show that the absolute, relative and parsimonious fit indices are all good, i.e., the model achieves good fitness.

**Table 3:** Analysis of the suitability.

**Category**	**Absolute fit index**			**Relative fit index**		**Parsimonious normed fit index**		
**Index**	**RMR**	**GFI**	**RMSEA**	**CFI**	**NFI**	**PNFI**	**χ2/df**	**CN**
Criterion	<0.05	>0.90	<0.08	>0.90	>0.90	>0.50	<5	>200
Original mode	0.014	0.958	0.067	0.982	0.974	0.673	3.373	222
Fitness	O	O	O	O	O	O	O	O

*O: meet the criterion*

### The Full Mediating Role of CSR Between RDHL and OC

The key to achieving organizational sustainability is incorporating CSR into the organization's strategy and practice (Setthasakko, 2007). Through CSR, the key ELB, RDHL can make employees feel more valuable in their existence by maintaining co-development with others (Hargreaves and Goodson, 2004), and thus are willing to play an important role in development. the organization (Sharma et al., 2019).

**Figure 2:**
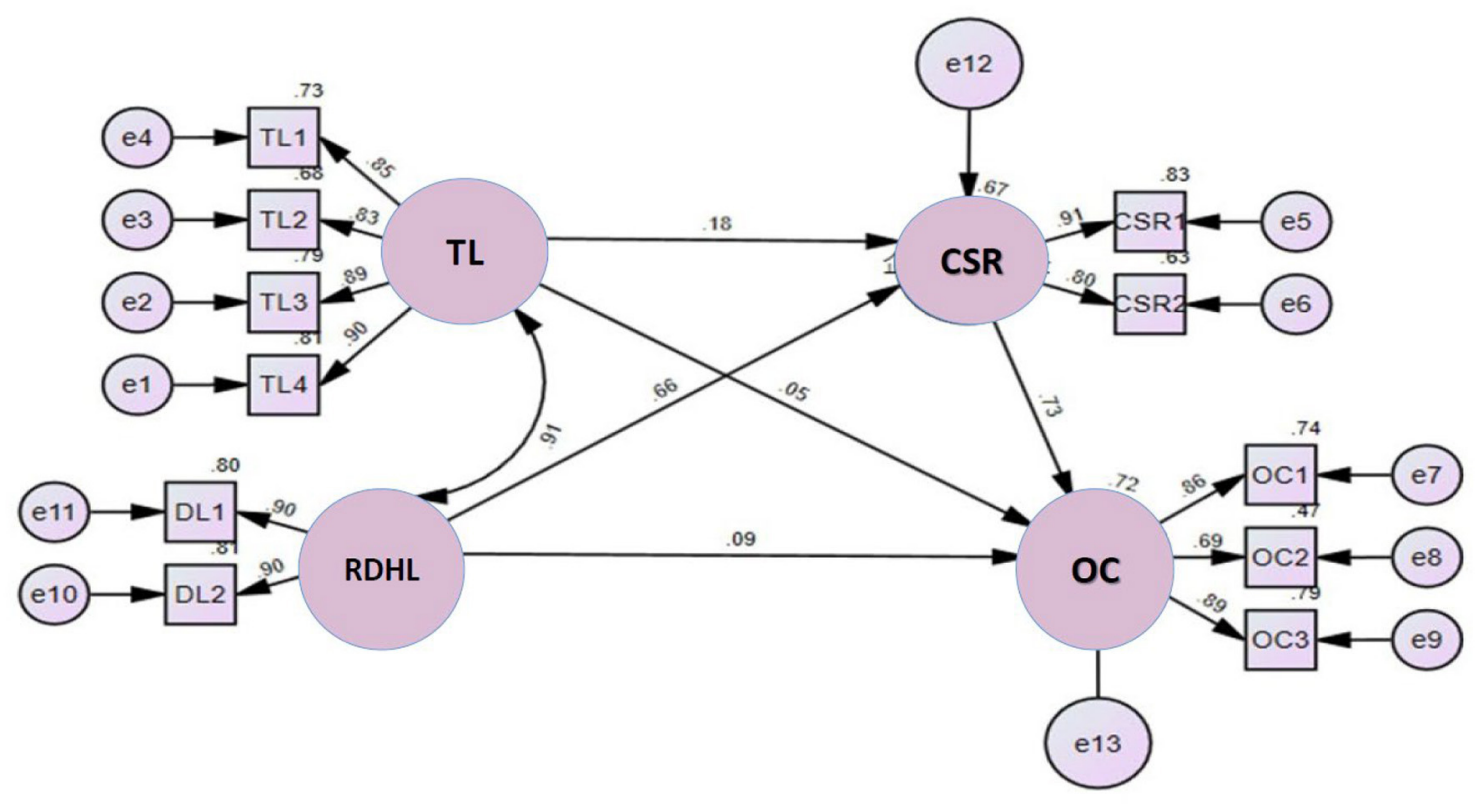
Path diagram of the SEM model.

The result of the model path coefficients in Figure 2 shows that though TL and RDHL have a correlation coefficient of 0.91, RDHL has a coefficient of 0.66 on CSR, which is significant and has a direct effect on the latter; though RDHL has a coefficient of 0.09 on OC, which is not significant and represent no direct effect, RDHL is through the CSR mediation model. CSR has a coefficient of 0.73 on OC, which is significant and represents a direct effect. The coefficient of influence of CSR on OC was 0.73, which was significant and represented a direct effect. The coefficient of influence of TL on CSR was 0.18, which was not significant, and the coefficient of influence of TL on OC was 0.05, which was also not significant and represented no direct effect. Therefore, the model of this study found that CSR was only a full mediator of RDHL and OC.

## Conclusions

Based on the findings, this study can reach the following conclusions: (1) the RDHL scale was well developed with qualified psychometrics evidence. (2) Although RDHL has a strong correlation with transformational leadership (TL), which is the most commonly used in past research when the two are put together in model testing, it can be found that TL has no predictive power on CSR and OC at all. However, RDHL does still have a fairly significant mediating effect on OC through CSR. Based on the above conclusions, this study triggers a more extensive theoretical dialogue in expanding the meaning of qualified leadership style during this innovative industry-four era. The following three sections have details presented: Practical Implications, Theoretical Implication & Research Limits and Suggestions for Future Studies.

### Practical Implications

Amid an organization's transformation from the present to the future sustainable model, leaders will face ongoing dilemmas of organizational conflicts and contradictions. However, at different stages of development, what kinds of leadership should be applied to create and achieve organizational sustainability in response to rapid change and constant challenges?

Firstly, one of the key findings of this research is that CSR is a full mediator between resource dilemmas in leadership (RDHL) and organizational commitment. The impact of CSR on OC should therefore be adopted through the influence of leadership on the external organizational performance of CSR to promote the internal strengthening of OC, in order to create and achieve the goal of organizational sustainability. Secondly, the training and enhancement of leadership skills can be improved based on the impact of RDHL in the future application of human resource management strategies and the training and succession of leaders, in order to create and realize sustainability.

As organizational sustainability refers to a long-term process, it is not easy to measure the effectiveness of an organization's sustainable development in terms of its duration. Thus, longitudinal research can be adopted in the future. Because TL's effect is found to be replaced by RDHL in the model, there are several extra recommendations for future research. Firstly, the findings of this study should be extrapolated with caution. Future research and comparisons could be conducted using different methods, such as longitudinal studies or cross-industry comparisons. Secondly, though the RDHL scale has reliability and validity due to its questions generalized from qualitative and case studies, additional questions can be further constructed, improved and developed in the future to enhance the applicability and usefulness of the scale, in order to enhance its practical applicability.

This study found that: RDHL affects organizational commitment through CSR's mediation. And the impact of RDHL on CSR is greater than the impact of TL on CSR, which means that RDHL plays a significant role in organizational commitment through its connection with enterprises' concerns and pursuit of CSR. This also proves that RDHL is not an additive capacity for TL, but rather a basis for comparison. This study used the questionnaire to test the impact of TL and RDHL on CSR and OC and found that RDHL can replace existing variable TL and has better performance in handling dilemma resources in CSR and OC performance in such a requesting-sustainable-resources era.

Moreover, the authors of this study believe that CSR itself is like a sense of responsibility, giving employees a sense of mission. Because of attaching importance to the sense of mission, employees feel more meaningful and able to complete the tasks assigned by the organization, resulting in OC. So, this study innovatively proposes a new theoretical concept that this is like a hatchery, allowing employees to gain a sense of accomplishment in learning the meaning of the mission and completing meaningful tasks in the organization, which could be more prominent in NPO organizations. For this study, through the CSR's hatchery in multiple emotional senses, employees are regarding CSR as ELB during the process of sustainable development. On the other hand, this phenomenon should be able to bring some practical implications for NPOs.

### Theoretical Implications

The most important significance and capability of RDHL leadership is the ability to create a sustainable future for the organization through leadership integration or reform in the face of rapid changes and unpredictable challenges in the future, as principles suggested by Efthimiou (2017).

First, this article extensively contributes to explaining the ELB concept with the variable of CSR. If CSR is viewed as an ELB, employees' recognition of CSR beliefs will become an important mediator for promoting organizational commitment. This may have indirectly proved that employees are willing to invest in organizational commitment because they identify with the leader's effective beliefs (CSR in this study). While TL has good talents in the organization's transformation, it has some beliefs-focused missions, such as sustainable development of the organization that will need the appearance of ELB. At this point, careful consideration of the ELB is an important thing. Rus et al. (2010) explored the concept of ELB and leader resource allocations. This study theoretically contributes to extending Rus et al. theoretical dialogue in taking CSR as one of the key ELBs to utilize for RDHL during the process of sustainable development. This is supported by the fact that the SEM examination of the impact of leadership and organizational sustainability revealed that RDHL exerted influence on OC through CSR. CSR only had a full mediating effect between RDHL and OC. The empirical findings of this study showed that CSR had a mediating effect between leadership and OC, which is consistent with previous research (Farooq et al., 2014; George et al., 2020). Therefore, the impact of leadership on organizational sustainability, through the impact of RDHL on the performance of CSR outside the organization, will help to enhance employees' recognition of the organization's social image and reputation, thereby generating an impact on the energy of organizational commitment within the organization to create and achieve the goal of organizational sustainability.

Secondly, this research found RDHL [based on Havenga et al. (2011) proposed to integrate three major leadership theories to deal with corporate resources dilemma] had a strong correlation with the transformational leadership, which had been most frequently applied to understand the relationship between leadership and sustainability in past research. Though RDHL and TL are highly correlated, they are still not the same, referring to the findings. The former is more influential than the latter in terms of its impact on organizational sustainability. In previous studies, TL has been shown to have a positive impact on employee sustainability performance (Jiang et al., 2017), to play a significant role in the development of core competencies sustaining an organization's sustainability (Choi, 2016), and to have a direct impact on the sustainability goals achieved by the organization (Tabassi et al., 2016).

Thirdly, the findings of this study are inconsistent with the findings regarding the impact of TL on organizational sustainability. In SEM model, RDHL replaced TL as a critical leadership force in creating sustainability in organizations. After verifying the SEM model, this study also conducted a regression verification of transformational leadership, CSR and OC, and found that the results were significant as the authors' proposal. This is consistent with previous findings in McMurray et al., 2010; Patiar and Wang, 2016; Tabassi et al., 2016; Allen et al., 2017; Khan et al., 2018; Palalic and Ait Sidi Mhamed, 2020). Since RDHL's practice of CSR provides a basis for organizational sustainability (Allen et al., 2017); it also positively impacts employee commitment and organizational evaluation (McMurray et al., 2010).

From this, it can be seen that RDHL is more influential in predicting corporate sustainability compared with TL. In other words, RDHL does not contradict the results of previous studies, which suggested that TL has significant predictive power on corporate sustainability. This is the first time that such a phenomenon has been explored in deeper aspects, and it should continue to be discussed in the future.

**Figure 3:**
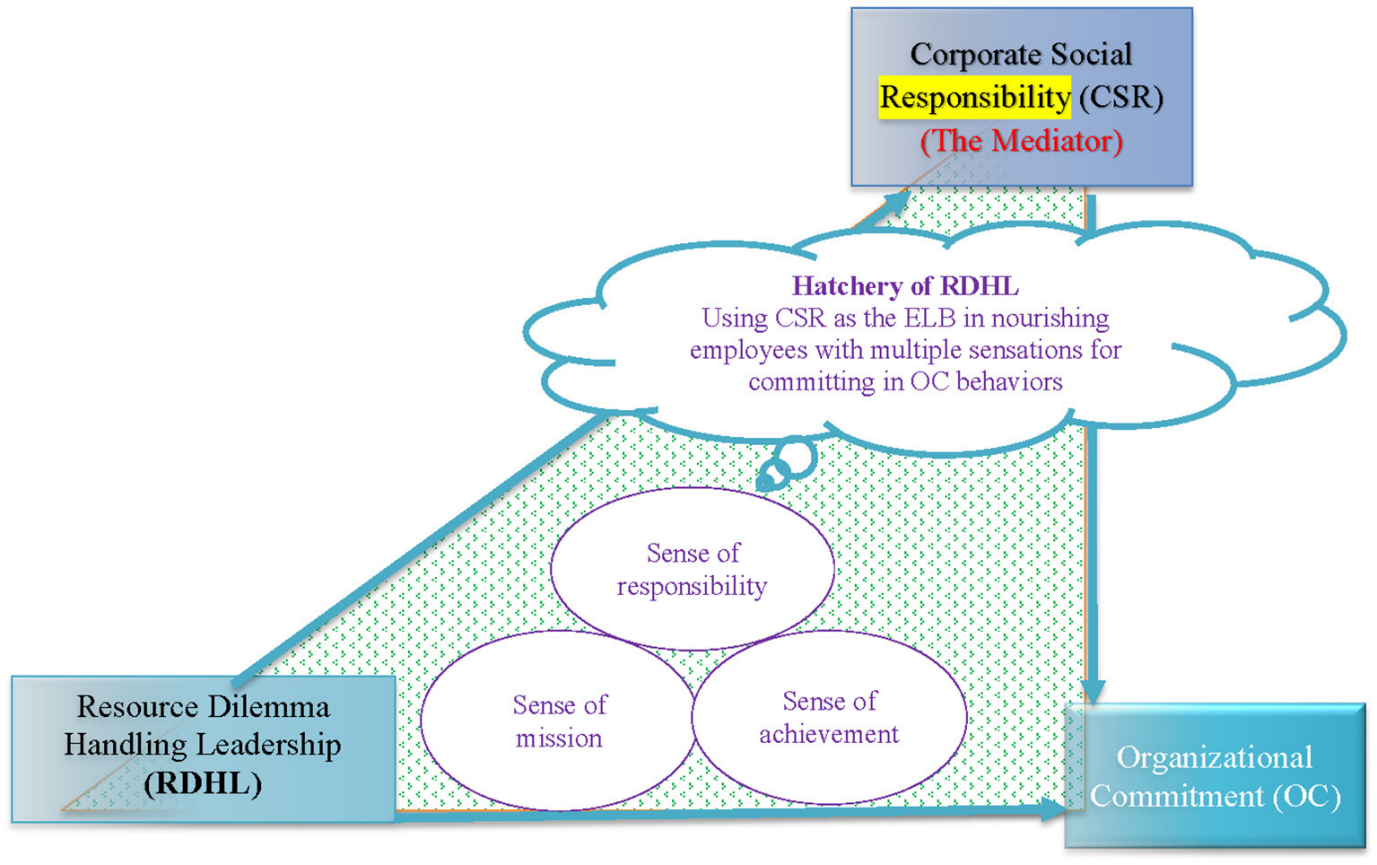
Through the CSR's hatchery in multiple emotional senses, employees regard CSR as ELB during the process of sustainable development. CSR itself is like a sense of responsibility, giving employees a sense of mission. Because of attaching importance to the sense of mission, employees feel more meaningful and better e to complete the tasks assigned by the organization, resulting in OC. It is like a hatchery, allowing employees to gain a sense of accomplishment in learning the meaning of the mission and completing meaningful tasks in the organization.

In short, it is worthwhile to continue to explore the impact of RDHL on leadership and organizational sustainability in the future. Therefore, this study suggested that RDHL, CSR, and OC could be organizational critical and supportive organizational behaviors in supporting the commitment to sustainable development (Figure 3). As indicated in Figure 3, RDHL, CSR, and OC form foundations in supporting the triangle of commitment to sustainable development (Figure 3), which deserves extensive investigation in the future. As indicated in Figure 3, RDHL, CSR, and OC form foundations in supporting the triangle of commitment to sustainable development.

### Research Limits and Suggestions for Future Studies

This study firstly contributes to propose (1) a quick comparison between TL and RDHL's influence level upon CSR, as well as the potential impact on enterprises' taking responsibility and commitment to SDG and sustainable development in the long run; (2) raise a new concept, which is RDHL, and thus expect to arise the possibility to incrementally require capability promotion of leaders in responding to the demands of sustainability development. However, this is the first time that ever a study raises this type of concept, RDHL, which requires repetitive validation on the development of this variable.

This study found that RDHL and TL are different but highly correlated. TL is the leadership style proposed early on, successfully assisting the organization to face challenges and help the organization change, transform, and adapt to the external environment. However, within the discussion of external environment development, the scarcity of resources, which needs to be protected and sustainably developed, has become an important issue and needs to be integrated into the development of leadership, which is RDHL discussed in this study. But these two leadership styles are not in conflict with each other, but can supplement each other. Therefore, the authors suggest that enterprises should start upgrading and developing a new leadership style that fits into new customers' expectations toward enterprises in terms of CSR and organizational commitment to sustainable development and concerns. For example, new RDHL leaders should have more concerns about protecting environmental resources when considering the input materials, saving energy when designing g production procedures, or even saving materials when packing and delivering products to customers. Therefore, workforce development, from TL to RDHL in terms of importance, procedures, significant benefits, and RDHL leaders' motivation toward employees in terms of sustainable development becomes future suggested studies made by the authors.

## Data Availability Statement

The original contributions presented in the study are included in the article/supplementary material, further inquiries can be directed to the corresponding author.

## Author Contributions

K-HC and I-TS is in charge of conceptual research framework of this study and supervising on the progress of the research. S-PL for the literature review and conclusions' theoretical and practical implications. All authors contributed to the article and approved the submitted version.

## Conflict of Interest

The authors declare that the research was conducted in the absence of any commercial or financial relationships that could be construed as a potential conflict of interest.

## Publisher's Note

All claims expressed in this article are solely those of the authors and do not necessarily represent those of their affiliated organizations, or those of the publisher, the editors and the reviewers. Any product that may be evaluated in this article, or claim that may be made by its manufacturer, is not guaranteed or endorsed by the publisher.

## Appendix

**Appendix A:** RDHL Questionnaire Items developed by this study.

**Item**	**Please circle the appropriate answer based on your true feelings**
1	The supervisor has made long-term and substantial investments to support the innovative behavior of employees, while at the same time improving the company's short-term operational efficiency.
2	The supervisor values the cost-effectiveness of his own time, while at the same time communicating and sharing information internally at a higher cost of time.
3	The supervisor is very concerned about the control of HR cost effectiveness, while at the same time not hesitating to pay higher personnel costs in the appointment and management of diversity of employees by gender, age, race, nationality, religion, etc.
4	The supervisor is committed to the interests and well-being of the community, while at the same time not harming the company's interests.
5	The supervisor is very concerned about the revenue performance of the company, while at the same time not hesitating to pay higher capital costs and invest in environmental protection.
6	The supervisor is very concerned about the financial performance of the company's operations, while at the same time helping employees to develop their careers.
7	The supervisor places great importance on creating company benefits, while at the same time sharing the operational results with employees.
8	The supervisor can be “strict with himself/herself,” while at the same time being “generous to others.”
9	The supervisor values the company's unique development and niche, while at the same time respecting and adopting the opinions of employees.
10	The supervisor attaches great importance to the establishment and transmission of the organization's traditional culture, while at the same time leading the company to pursue breakthroughs and innovations.
11	The supervisor will invest heavily in enhancing company's core business strength, while at the same time making a high-cost contribution to the physical and mental growth and care of their employees.
12	The supervisor is very concerned about the company's business growth, while at the same time providing more open and transparent information on product transactions to consumers.
13	The supervisor will highly cooperate with government regulations and promote the policy implementation, while at the same time generating revenue performance for the company.

## References

[B1] Allen G. W. Attoh P. A. Gong T. (2017). Transformational leadership and affective organizational commitment: mediating roles of perceived social responsibility and organizational identification. Soc. Responsib. J. 13, 585–600. 10.1108/SRJ-11-2016-0193

[B2] Avissar I. Alkaher I. Gan D. (2018). The role of distributed leadership in mainstreaming environmental sustainability into campus life in an Israeli teaching college: a case study. Int. J. Sustain. High. Educ. 19, 518–546. 10.1108/IJSHE-07-2017-0105

[B3] Bass B. M. Avolio B. J. (2000). MLQ Multifactor Leadership Questionnaire, Seconded. Mind garden, Redwood City, CA.

[B4] Bendell J. Sutherland N. Little R. (2017). Beyond unsustainable leadership: critical social theory for sustainable leadership. Sustain. Account. Manag. Policy J. 8, 418–444. 10.1108/SAMPJ-08-2016-0048

[B5] Çalişkan H. K. (2015). Technological change and economic growth. Procedia Soc. Behav. Sci. 195, 649–654. 10.1016/j.sbspro.2015.06.174

[B6] Choi M. (2016). Leadership of information security manager on the effectiveness of information systems security for secure sustainable computing. Sustainability. 8, 638. 10.3390/su8070638

[B7] Dreachslin J. L. Weech-Maldonado R. Gail J. Epane J. P. Wainio J. A. (2017). Blueprint for sustainable change in diversity management and cultural competence: Lessons from the national center for healthcare leadership diversity demonstration project. J. Healthc. Manag. 62, 171–183. 10.1097/JHM-D-15-00029 28471853

[B8] Efthimiou O. (2017). Heroic ecologies: embodied heroic leadership and sustainable futures. Sustain. Account. Manag. Policy J. 8, 489–511. 10.1108/SAMPJ-08-2015-0074

[B9] Fan S. C. Shih H. C. Tseng H. C. Chang K. F. Li W. C. Shia A. S. (2021). Self-efficacy triggers psychological appraisal mechanism for mindset shift. Int. J. Ment. Health Promot. 23, 57–73. 10.32604/IJMHP.2021.012177

[B10] Farooq O. Payaud M. Merunka D. (2014). The impact of corporate social responsibility on organizational commitment: exploring multiple mediation mechanisms. J. Bus. Ethics. 125, 563–580. 10.1007/s10551-013-1928-3

[B11] Fenwick T. (2007). Developing organizational practices of ecological sustainability: a learning perspective. Leadersh. Organ. Dev. J. 28, 632–645. 10.1108/01437730710823888

[B12] Ferrari J. R. (2004). Australian eldercare providers: comparing volunteers and temporary staff on work environment, interpersonal relationships, and self-efficacy. Eval. Health Prof. 27, 383–397. 10.1177/0163278704270008 15492049

[B13] George N. A. Aboobaker N. Edward M. (2020). Corporate social responsibility, organizational trust and commitment: a moderated mediation model. Pers. Rev. 50, 1093–1111. 10.1108/PR-03-2020-0144

[B14] Glavas A. (2016). Corporate social responsibility and organizational psychology: an integrative review. Front. Psychol. 7, 144. 10.3389/fpsyg.2016.00144 26909055 PMC4754563

[B15] Global Reporting Initiative (2006). Sustainability Reporting Guidelines Version 3.0 (GRI G3). Available online at: http://www.globalreporting.org/Home/GRI

[B16] Gupta M. (2017). Corporate social responsibility, employee–company identification, and organizational commitment: mediation by employee engagement. Curr. Psychol. 36, 101–109. 10.1007/s12144-015-9389-834305697

[B17] Hair J. F. Black W. C. Babin B. J. Anderson R. E. (2009). Multivariate Data Analysis. 7th, Upper Saddle River, NJ: Prentice-Hall.

[B18] Haque A. Fernando M. Caputi P. (2021). How is responsible leadership related to the three-component model of organizational commitment? Int. J. Product. Perform. Manag. 70, 1137–1161. 10.1108/IJPPM-10-2019-0486

[B19] Hargreaves A. Fink D. (2000). The three dimensions of education reform. Educ. Leadersh. 57, 30–34.

[B20] Hargreaves A. Fink D. (2004). The seven principles of sustainable leadership. Educ. Leadership. 61, 8–13.

[B21] Hargreaves A. Goodson I. (2004). Change Over Time? A Report of Educational Change Over 30 Years in Eight U.S. and Canadian Schools. Spencer Foundation: Chicago.

[B22] Harris J. M. (2003). Sustainability and sustainable development. Int. S. Ecol. Econ. 1, 1–12.

[B23] Havenga W. Mehana V. Visagie J. C. (2011). Developing a national cadre of effective leadership towards sustainable quality service delivery in South Africa. Afr. J. Bus. Manag. 5, 12271–12282. 10.5897/AJBM11.451

[B24] Horlings I. Padt F. (2013). Leadership for sustainable regional development in rural Areas: Bridging personal and institutional aspects. Sustain. Dev. 21, 413–424. 10.1002/sd.526

[B25] Hwang I.-S. Chi D.-J. (2005). A study on enterprise ethics, social responsibility and corporate philanthropy- taking the hi-tech electronic industry in Taiwan as an example. J. Human. Soc. Sci. 1, 65–82.

[B26] Jaros S. (2007). Meyer and allen model of organizational commitment: measurement issues. The ICFAI J. Organ. Behav. 6, 7–25.

[B27] Jiang W. P. Zhao X. B. Ni J. B. (2017). The impact of transformational leadership on employee sustainable performance: the mediating role of organizational citizenship behavior. Sustainability. 9, 1567. 10.3390/su9091567

[B28] Jones D. A. Willness C. R. Glavas A. (2017a). When corporate social responsibility (CSR) meets organizational psychology: new frontiers in micro-CSR research, and fulfilling a quid pro quo through multilevel insights. Front. Psychol. 8, 520. 10.3389/fpsyg.2017.00520 28439247 PMC5383697

[B29] Jones S. A. Michelfelder D. Nair I. (2017b). Engineering managers and sustainable systems: the need for and challenges of using an ethical framework for transformative leadership. J. Clean. Prod. 140, 205–212. 10.1016/j.jclepro.2015.02.009

[B30] Joo B. Yoon H. Jeung C. (2012). The effects of core self-evaluations and transformational leadership on organizational commitment. Leadersh. Organ. Dev. J. 33, 564–582. 10.1108/01437731211253028

[B31] Joo B. K. Lim T. (2009). The effects of organizational learning culture, perceived job complexity, and proactive personality on organizational commitment and intrinsic motivation. J. Leadersh. Organ. Stud. 16, 48–60. 10.1177/1548051809334195

[B32] Kaiser H. F. (1974). An index of factorial simplicity. Psychometric 39, 31–36. 10.1007/BF02291575

[B33] Khan H. U. R. Ali M. Olya H. G. Zulqarnain M. Khan Z. R. (2018). Transformational leadership, corporate social responsibility, organizational innovation, and organizational performance: symmetrical and asymmetrical analytical approaches. Corp. Soc. Responsib. Environ. Manag. 25, 1270–1283. 10.1002/csr.1637

[B34] Kono N. (2014). Brundtland Commission (World Commission on Environment and Development), in Encyclopedia of Quality of Life and Well-Being Research, ed. Michalos A. C. (Springer, Dordrecht). 10.1007/978-94-007-0753-5_441

[B35] Kurucz E. C. Colbert B. A. Ludeke-Freund F. Upward A. Willard B. (2017). Relational leadership for strategic sustainability: Practices and capabilities to advance the design and assessment of sustainable business models. J. Clean. Prod. 140, 189–204. 10.1016/j.jclepro.2016.03.087

[B36] Landrum N. E. Edwards S. (2012). A Primer on Sustainable Business. Irvington, NY: Faculty Books, 35.

[B37] Lee K. H. Schaltegger S. (2014). Organizational transformation and higher sustainability management education: the case of the MBA sustainability management. Int. J. Sustain. Higher Educ. 15, 450–472. 10.1108/IJSHE-06-2013-0067

[B38] Lord R. G. Maher K. J. (1993). Leadership and Information Processing: Linking Perceptions and Performance. London: Rutledge.

[B39] Lukoschek C. S. Gerlach G. Stock R. M. Xin K. (2018). Leading to sustainable organizational unit performance: antecedents and outcomes of executives' dual innovation leadership. J. Bus. Res. 91, 266–276. 10.1016/j.jbusres.2018.07.003

[B40] Mallén Broch F. F. Domínguez Escrig E. Chiva Gómez R. Lapiedra Alcam,í R. (2020). Promoting firm innovativeness through servant leadership and corporate social responsibility to employees. Leadersh. Organ. Dev. J. 41, 615–633. 10.1108/LODJ-03-2019-0127

[B41] Mayo M. Gomez-Mejia L. Firfiray S. Berrone P. Villena V. H. (2016). Leader beliefs and CSR for employees: the case of telework provision. Leadersh. Organ. Dev. J. 37, 609–634. 10.1108/LODJ-09-2014-0177

[B42] Mazutis D. Zintel C. (2015). Leadership and corporate responsibility: a review of the empirical evidence. Annals Soc. Res. 1, 76–107. 10.1108/ASR-12-2014-0001

[B43] McCann J. Sweet M. (2014). The perceptions of ethical and sustainable leadership. J. Bus. Ethics. 121, 373–383. 10.1007/s10551-013-1704-4

[B44] McMurray A. J. Pirola-Merlo A. Sarros J. C. Islam M. M. (2010). Leadership, climate, psychological capital, commitment, and wellbeing in a non-profit organization. Leadersh. Organ. Dev. J. 31, 436–457. 10.1108/01437731011056452

[B45] Meindl J. R. Ehrlich S. B. (1987). The romance of leadership and the evaluation of organizational performance. Acad. Manag. J. 30, 91–109. 10.5465/255897

[B46] Meyer J. Allen N. (1997). Commitment in the Workplace: Theory, Research, and Application. Thousand Oaks, CA: Sage Publications. 10.4135/9781452231556

[B47] Najam U. Inam A. Awan H. M. Abbas M. (2018). The Interactive role of temporal team leadership in the telecom sector of Pakistan: utilizing temporal diversity for sustainable knowledge sharing. Sustainability. 10, 1309. 10.3390/su10051309

[B48] Nguyen H. T. Le D. M. D. Ho T. T. M. Nguyen P. M. (2021). Enhancing sustainability in the contemporary model of CSR: a case of fast fashion industry in developing countries. Soc. Responsib. J. 17, 578–591. 10.1108/SRJ-03-2019-0108

[B49] Palalic R. Ait Sidi Mhamed E. M. (2020). Transformational leadership and MNCs: evidence from Morocco community. J. Enterp. Comm.: People and Places in the Global Economy. 14, 201–230. 10.1108/JEC-01-2020-0002

[B50] Paruzel A. Klug H. J. P. Maier G. W. (2021). The relationship bet‘en perceived corporate social responsibility and employee-related outcomes: a meta-analysis. Front. Psychol. 12, 607108. 10.3389/fpsyg.2021.607108 34305697 PMC8295475

[B51] Patiar A. Wang Y. (2016). The effects of transformational leadership and organizational commitment on hotel departmental performance. Int. J. Contemp. Hosp. Manag. 28, 586–608. 10.1108/IJCHM-01-2014-0050

[B52] Rus D. Van Knippenberg D. Wisse B. (2010). Leader power and leader self-serving behavior: the role of effective leadership beliefs and performance information. J. Exp. Soc. Psychol. 46, 922–933. 10.1016/j.jesp.2010.06.007

[B53] Schnaider T. B. Silva J. V. D. Pereira M. A. D. R. (2009). Family care providers of neurologically affected patients. Saúde e Sociedade. 18, 284–292. 10.1590/S0104-12902009000200011

[B54] Setthasakko W. (2007). Determinants of corporate sustainability: Thai frozen seafood processors. Br. Food J. 109, 155–168. 10.1108/00070700710725518

[B55] Sharma A. Agrawal R. Khandelwal U. (2019). Developing ethical leadership for business organizations. Leadersh. Organ. Dev. J. 40, 712–734. 10.1108/LODJ-10-2018-0367

[B56] Shriberg M. MacDonald L. (2013). Sustainability leadership programs: Emerging goals, methods and best practices. J. Environ. Educ. 5. 10.1016/S0952-8733(02)00006-5

[B57] Suriyankietkaew S. Avery G. (2016). Sustainable leadership practices driving financial performance: empirical evidence from Thai SMEs. Sustainability. 8, 327. 10.3390/su8040327

[B58] Tabassi A. A. Roufechaei K. M. Ramli M. Bakar A. H. A. Ismail R. Pakir A. H. K. (2016). Leadership competences of sustainable construction project managers. J. Clean. Prod. 124, 339–349. 10.1016/j.jclepro.2016.02.076

[B59] Taskin L. Devos V. (2005). Paradoxes from the individualization of human resource management: The case of telework. J. Bus. Ethics. 62, 13–24. 10.1007/s10551-005-8710-0

[B60] Tilleman S. G. (2012). Is employee organizational commitment related to firm environmental sustainability? J. Small Bus. Entrepreneurship 0.25, 417–431. 10.1080/08276331.2012.10593582

[B61] van Gils S. van Quaquebeke N. van Knippenberg D. (2010). The X-factor: on the relevance of implicit leadership and followership theories for leader–member exchange agreement. Eur. J. Work Organ. Psychol. 19, 333–363. 10.1080/13594320902978458

[B62] Wang X.-H. F. Yang J. Cao R. Lee B. Y. (2019). Corporate social responsibility and collective OCB: A social identification perspective. Front. Psychol. 10, 2720. 10.3389/fpsyg.2019.02720 31920789 PMC6917590

[B63] Weber T. J. Sadri G. Gentry W. A. (2018). Examining diversity beliefs and leader performance across cultures. Cross Cult. Strateg. Manag. 25, 382–400. 10.1108/CCSM-11-2016-0200

[B64] West S. G. Finch J. F. Curran P. J. (1995). “Structural equation models with normal variables: Problems and remedies”, in Structural equation modeling: Concepts, issues and applications, ed. R. Hoyle Newbury Park (CA: Sage).

